# Microglia Diversity in Health and Multiple Sclerosis

**DOI:** 10.3389/fimmu.2020.588021

**Published:** 2020-11-06

**Authors:** Sameera Zia, Khalil S. Rawji, Nathan J. Michaels, Mena Burr, Bradley J. Kerr, Luke M. Healy, Jason R. Plemel

**Affiliations:** ^1^ Neuroscience and Mental Health Institute, University of Alberta, Edmonton, AB, Canada; ^2^ Wellcome Trust-Medical Research Council Cambridge Stem Cell Institute, Jeffrey Cheah Biomedical Campus, Cambridge Biomedical Campus, University of Cambridge, Cambridge, United Kingdom; ^3^ Ministry of Health, British Columbia Government, Victoria, BC, Canada; ^4^ Department of Anesthesiology & Pain Medicine, University of Alberta, Edmonton, AB, Canada; ^5^ Neuroimmunology Unit, Department of Neurology and Neurosurgery, Montreal Neurological Institute and Hospital, McGill University, Montreal, QC, Canada; ^6^ Department of Medicine, Division of Neurology, University of Alberta, Edmonton, AB, Canada

**Keywords:** microglia, macrophages, single-cell analysis, single-cell RNA sequencing, multiple sclerosis, remyelination, ageing

## Abstract

Multiple Sclerosis (MS) is a neurodegenerative disease characterized by multiple focal lesions, ongoing demyelination and, for most people, a lack of remyelination. MS lesions are enriched with monocyte-derived macrophages and brain-resident microglia that, together, are likely responsible for much of the immune-mediated neurotoxicity. However, microglia and macrophage also have documented neuroprotective and regenerative roles, suggesting a potential diversity in their functions. Linked with microglial functional diversity, they take on diverse phenotypes developmentally, regionally and across disease conditions. Advances in technologies such as single-cell RNA sequencing and mass cytometry of immune cells has led to dramatic developments in understanding the phenotypic changes of microglia and macrophages. This review highlights the origins of microglia, their heterogeneity throughout normal ageing and their contribution to pathology and repair, with a specific focus on autoimmunity and MS. As phenotype dictates function, the emerging heterogeneity of microglia and macrophage populations in MS offers new insights into the potential immune mechanisms that result in inflammation and regeneration.

## Introduction

Microglia are a specialized population of myeloid cells in the brain and spinal cord, and depending on the species and anatomical region, account for 0.5–16.6% of total central nervous system (CNS) cells (1, 2). Under homeostatic conditions they are the primary macrophage-like cell in the CNS. To maintain homeostasis microglia act as sentinels, continually surveying their environment by extending and retracting their motile processes, ready to respond to the first signs of pathogenic invasion or tissue damage (3). In the event of inflammation, microglia help orchestrate the immune response, balancing the risk of potential harm to delicate CNS tissue and supporting tissue repair and remodeling. The central role of microglia in the defense and maintenance of the brain and spinal cord implicates them in nearly all brain pathologies (4).

Microglia are derived from the embryonic yolk sac and take residence in the CNS early in development (3). As the brain and spinal cord mature, microglia respond to the changing environment and, help shape CNS tissue development; microglia contribute to the remodeling of postnatal neural circuits and play a role in synaptic pruning during postnatal development (5, 6). This bidirectional communication with the CNS during development helps establish a unique microglial identity. Once established, microglia density is sustained by balancing microglia proliferation and cell death, without a contribution from blood-derived cells (7).

In the last decade, advancements in technologies such as single-cell RNA sequencing and lineage tracing has shed light on the way microglia function under steady state conditions and during disease. Lineage tracing and genetic fate mapping allow microglia to be distinguished from other macrophage-like cells, which becomes crucial during pathological conditions as monocyte-derived cells enter the CNS parenchyma from the periphery and the two cell types become virtually indistinguishable from one another using classical markers (8). Techniques such as MARS-Seq and Drop-Seq, among many others, allow gene expression to be analyzed at the single-cell level. The ability to focus on microglia explicitly, combined with single-cell sequencing has allowed greater insight into cell trajectories, cell states, gene networks, and receptor-ligand interactions. This information supplements what is known about microglia across the lifespan, during development and during disease, including autoimmune demyelinating disorders such as multiple sclerosis (MS).

The pathological hallmark of MS is the formation of demyelinating lesions in the brain and spinal cord (9). These focal lesions are ubiquitously associated with the infiltration and activation of immune cells. Microglia are among the first responders and remain within lesions until the lesion resolves or becomes inactive. The lesion microenvironment changes over time and differs with anatomical location—i.e. white matter versus grey matter. The presence or absence of remyelination further complicates the lesion environment. Microglia are influenced by these changing lesion environments and are tasked with responding to the associated complex immune milieu. Understanding various microglia functions in MS lesions may help develop therapeutic interventions that tip the scale of the immune response towards repair and regeneration and away from tissue damage.

In this review, we discuss what is known about microglia origin and development; similarities and differences between human and murine microglia; and microglia heterogeneity throughout life, in the context of CNS autoimmunity and during remyelination and ageing. The heterogeneity of microglia during development, across the lifespan and in MS offers new insights into the potential immune mechanisms resulting in tissue inflammation or tissue regeneration.

## Microglia Establishment in the CNS

Microglia are CNS resident macrophages of the mononuclear phagocyte system (10). Under steady-state conditions, they are the primary resident myeloid population in the brain and spinal cord. Microglia first appear in early development (~E9.5 days post-conception) from a population of primitive macrophages that mature from mesodermal erythromyeloid progenitors in the embryonic yolk sac (11, 12). These primitive macrophages do not require the transcription factor Myb for their development, unlike monocyte-derived macrophages and those of the hematopoietic stem cell lineage (13). Initially, the primitive macrophages that give rise to microglia lack the classic leukocyte marker (Cd45) and express the receptor tyrosine kinase C-kit. They progressively lose C-kit expression while gaining expression of Cd45 as they mature (12, 14). These cells migrate through the developing vasculature to the brain rudiment, where they differentiate into microglia (13, 15). This migration starts around E9.5 in mice. Once inside the CNS, microglia undergo extensive local proliferation and spread out to populate the entire developing brain, ultimately acquiring their unique identity in tandem with neural tissue development (16).

Murine microglia isolated from various life stages reveal a progressive change in gene expression pattern that occurs in parallel with the developing brain as they influence and adapt to the changing CNS environment (17, 18). This reciprocal interaction between the developing brain and the maturing microglia population heavily influences the establishment of a unique microglia identity. Microglia identity is driven, in large part, by the activity of the critical lineage dependent transcription factors, Pu.1 and C/ebp (19). Mice lacking Pu.1 do not develop a microglia population (13). Other critical regulators of microglia identity include signal-dependent transcription factors such as Maf, Mef2c, Sall1and Irf8 (15, 20–22).

Local CNS factors maintain a healthy microglia population. Signalling through the colony-stimulating factor 1 receptor (Csf1r) is vital for microglial survival in mice, both developmentally and throughout the lifespan (23). Csf1 and Il-34 are the two known ligands for Csf1r that are both found in the CNS. Interestingly, microglia in white matter, grey matter and from distinct brain regions differ in their reliance on either Il-34 or Csf1 (24, 25). In the mature mouse brain, Tgf-β is another key regulator of microglia identity through the activity of Smad transcription factors (26, 27). During embryonic and early postnatal development, where there are high levels of microglia proliferation, Tgf-β is also a crucial contributor (28). Following a burst of postnatal proliferation, microglia self-renew slowly in a stochastic manner where the processes of proliferation and apoptosis are tightly coupled (7). While the exact rate of turnover is yet to be agreed upon, it is apparent that there are different rates of microglia turnover depending on brain location (7, 29) where human microglia divide on an average of 4.2 years, but some may not divide for over 20 years (30). Mouse microglia turnover approximately every 15 months (31).

The CNS contains other immune cells that may regulate microglia function. Outside the CNS parenchyma resides several distinct myeloid cell populations including perivascular, meningeal and choroid plexus macrophages. These populations are collectively known as border associated macrophages (BAM) (32, 33) or CNS-associated macrophages (CAM) (34). Heterogeneity between and even within BAM populations has recently been uncovered and their roles in mediating immune cell entry and activation of T-cells investigated (32, 35). BAMs display heterogeneity with respect to the expression of antigen presentation genes appearing postnatally, suggesting that BAM diversity is primarily shaped after birth, in part under the influence of microbiome-derived stimuli (36). The interactions between BAM cells and parenchymal microglia remains to be studied. Other immune cell populations reside in the cerebrospinal fluid such as lymphocytes, dendritic cells, neutrophils and monocyte-derived cells (32). These and peripheral blood-associated immune cells infiltrate the parenchyma during injury and under disease conditions to affect microglia function (37). The extent to which these cells exert a remote influence on microglia activity remains to be fully understood.

## Murine Microglia Heterogeneity Throughout Life

The advent of new technologies has allowed the exploration of cell biology at the single-cell resolution. Previous genomic strategies such as bulk RNA sequencing were focused on investigating global gene expression changes. These bulk strategies measured the average gene expression across a population of cells, which presented significant limitations in cases where cell types are heterogenous or divided into several populations with potentially different functions (38). To overcome this, Tang and colleagues developed single-cell sequencing technologies that used a combination of PCR amplification and microarray tools (39). With the expansion of new tools such as, MARS-Seq (38), Drop-Seq (40), Smart-Seq (41), Smart-Seq2 (42), Cel-seq (43), CEL-Seq2 (44) and SCRB-Seq (45)—that have been reviewed extensively by others (46, 47)—it is now possible to determine the transcriptome of cells or nuclei at an individual cell level. The study of microglia with single-cell resolution has allowed significant advances with recent developments in bioinformatics (48), such as defining cell trajectories (49), deciphering cell states, constructing gene regulatory networks (50) and inferring receptor-ligand interactions (51, 52).

One important discovery from single cell transcriptomic work is the presence of different microglial populations that vary phenotypically across development and lifespan. Embryonic and postnatal development is characterized by several unique microglia populations not present in adults (53, 54). For example, at E14.5 there is a population of metabolically active microglia enriched with lactate dehydrogenase (*Ldha*), an enzyme involved in glycolysis that produces lactate (53). This population is also enriched with migration inhibitory factor (*Mif*), which is often associated with microglia during inflammation (8). These observations suggest an overlap between microglia populations in development with those found during inflammation. During development, microglia prune synapses, clear dead cells and regulate cell numbers (55), which may account for this microglial inflammatory signature.

During the transition from embryonic development into the early postnatal period, there is some phenotypic overlap in microglia populations (53, 54). A population of proliferative microglia are enriched during embryonic development and at early stages postnatally (53). Proliferative microglia were enriched with different cell cycle-related genes and were found in equal magnitudes at E14.5 and P4/5, but not at P30 (53), which parallels other work on the proliferation of microglia (7). These proliferative microglia express genes related to the DNA damage response (*Ankle1*, *Lig1*), histone mRNA decay (*Eri1*) and epigenetic function such as histones and chromatin modifiers (54), suggesting that proliferation is largely limited to the embryonic and early postnatal time points when the microglia population is established.

**Figure 1:**
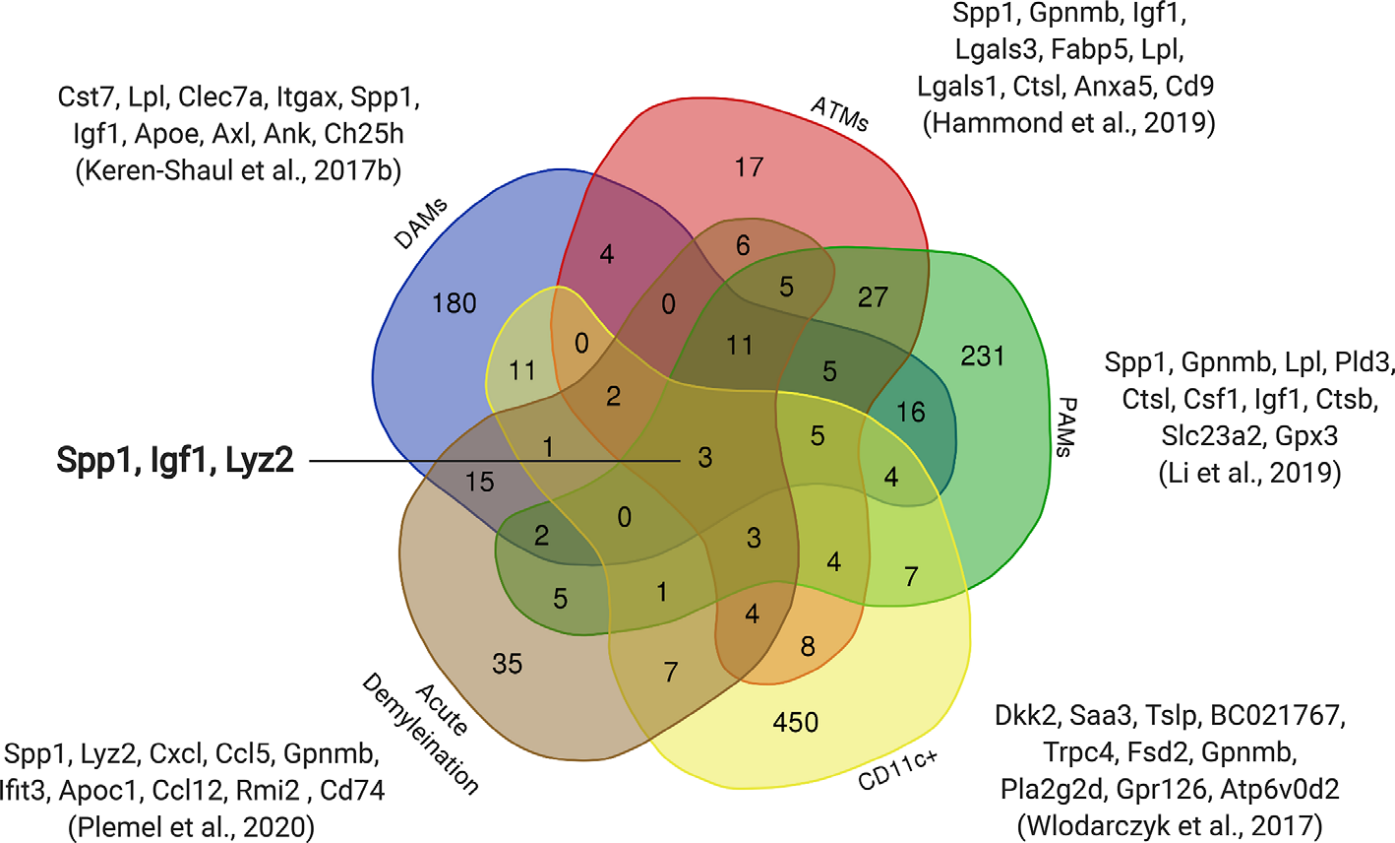
Overlap of upregulated genes between early postnatal microglia and microglia in diseased models. Subsets of early postnatal microglia (ATMs, PAMs, Cd11c+) with similar transcriptomic profiles were observed in three independent studies (53, 54, 58). The transcriptomic profile of an Alzheimer’s model (DAMs) (64) and an acute demyelination model (8) also show overlap with these postnatal microglia. The top ten upregulated genes from each dataset are shown with three genes that are common to all five datasets (*Spp1, Igf1, Lyz2*).

During the first three postnatal weeks another microglia population arises in developing white matter axonal tracts. This early postnatal period coincides with active myelination of the corpus callosum and cerebellum in mice (56, 57). Three independent groups have defined this interesting population of microglia. Wlodarczyk and colleagues found a population of microglia expressing Cd11c that are a significant source of Insulin-like growth factor 1 (Igf1) (58), an important survival factor that promotes myelin development (59). When *Igf1* was conditionally removed from these Cd11c microglial cells, there was reduced myelin gene expression (58), which is consistent with the finding that microglia regulate myelin development (60). Similarly, using single-cell RNA sequencing, Hammond and colleagues identified a population of microglia enriched in the developing axonal tracts they referred to as axonal tract microglia (ATM) (53). These ATMs were characterized by the distinct expression of genes related to lysosomal activation (*Lamp1*, *Cd68*) and possessed an amoeboid morphology (53). Microglia prune myelin sheaths in development (61), which may account for the amoeboid morphology and lysosomal activation characterizing ATM. Li and colleagues independently identified an equivalent population that they termed proliferative-region-associated microglia (PAM) (54). These amoeboid PAMs preferentially phagocytosed fluorescently labelled beads relative to other microglia phenotypes (54). PAMs were found to engulf newly formed oligodendrocytes, which incur significant cell death upon the onset of CNS myelination (62). The emergence of the PAM phenotype coincides with myelination onset and, therefore, may play an essential role in clearing the overproduced oligodendrocytes (63). PAMs also upregulated genes associated with lipid metabolism, lipid transport and lysosomal acidification, presumably necessitated by the phagocytosis of lipid-rich oligodendrocytes (54). The CD11c (58), ATM (53) and PAM (54) all contained common distinguishing genes such as *Spp1, Igf1* and *Gpnmb*, suggesting these populations are the same. Although, a comparison of these populations is needed to confirm the extent of overlap (**Figure 1**).

**Figure 2:**
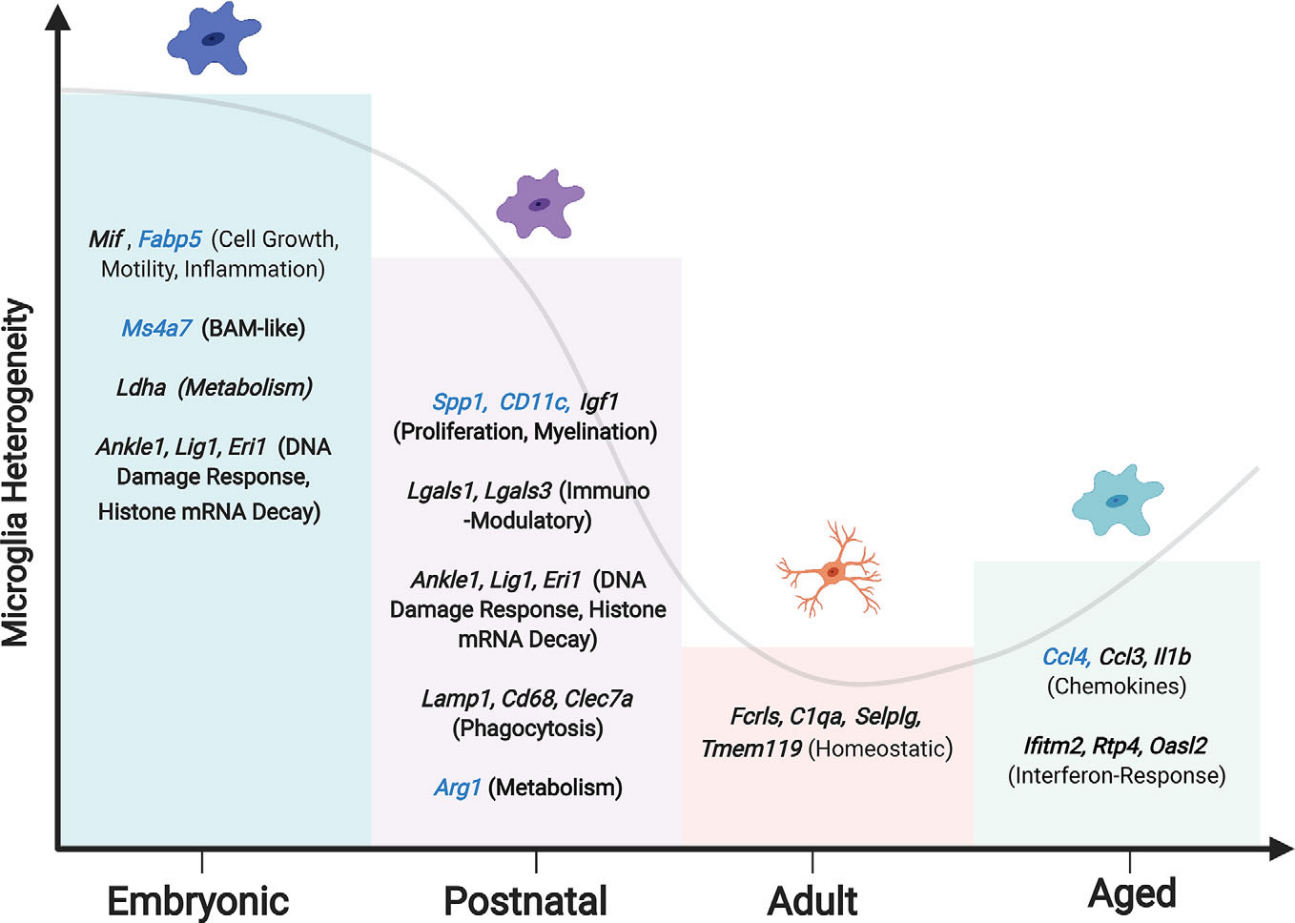
Changing microglia heterogeneity throughout development. Peak microglial heterogeneity is seen during embryonic development, with a decrease in adulthood, and a subsequent increase in the aged brain (65). Enriched genes and phenotypes significant to each microglial developmental stage are shown, with genes that are unique to each stage in blue. Hammond et al. reported a subset of embryonic microglia uniquely expressing *Ms4a7*, suggesting a similarity to BAMs.

Microglia diversity decreases after puberty, when microglia become more homogenous with fewer distinct phenotypes but with considerable variance in expression levels of homeostatic genes (**Figure 2**) (17, 53, 54). Adult homeostatic microglia are characterized by genes such as *Fcrls, Clqa, Selplg and Tmem119* (17, 27, 66, 67). Interestingly, the previously thought canonical microglia markers *(P2ry12, Cx3cr1, Tmem119)* are not found to be uniformly expressed across all homeostatic clusters and therefore may not be a robust way to detect microglia *in vivo* (53). The transition of microglia from the postnatal phenotype to the adult is dependent upon the transcription factor Mafb, without which microglia upregulate antiviral genes and lose their homeostatic nature (17).

**Figure 3:**
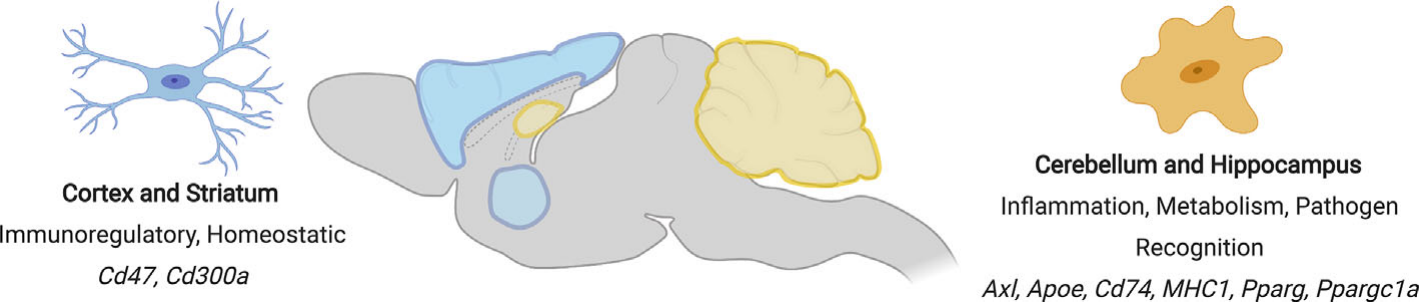
Regional differences in microglial phenotypes in the mouse brain. Regions showing similar microglial phenotypes are similarly coloured (cortex and striatum = blue, cerebellum and hippocampus = yellow). Phenotypic characteristics and signature genes for each region are shown.

Regional differences in microglia phenotypes may reflect their functional requirements (68). For example, the cerebellum has a high neuronal turnover rate compared to the striatum and has been found to house a microglia subset that appears to specialize in debris clearance and apoptotic cell detection (**Figure 3**). This subtype is characterized by the presence of the genes more commonly associated with inflammation, such as *Axl, Apoe, Cd74*, and MHC-I genes. As the striatum consists of a neuronal population that is relatively stable throughout adulthood, it does not require a phagocytic microglia phenotype and is therefore accompanied by a homeostatic microglia phenotype lacking expression of activation genes (69). The deep brain structures also showcase a distinct variety of microglia. Microglia in the basal ganglia nuclei differ in their densities, morphologies and electrophysical properties (70). In the ventral tegmental area of the basal ganglia, genes related to metabolism are depressed and those required for growth factor release and phagocytosis are upregulated.

Age-related changes to microglia populations occur and suggest an overall heightened inflammatory response with regional variability (68). While whole-brain analysis demonstrates a significant overlap between microglia populations in young and aged mice, there is an expansion of two microglia populations enriched in the aged brain (53). These age-associated microglia populations are either enriched in the chemokine *Ccl4*, lipoprotein lipase (*Lpl*) or genes associated with interferon response such as *Ifitm4, Ifit3*, and *Irf7*. With age, microglia accumulate myelin fragments within lysosomal structures (71), which likely account for new age-related microglial populations. The dominance of inflammatory subpopulations may contribute to progressive neurodegeneration, which is often age-dependent (53, 54, 72).

## Similarities Between Human and Murine Microglia

The similarities and differences between murine and human microglia have been explored in more detail elsewhere (27, 65). Here, we will briefly review recent work that has combined single-cell RNA sequencing with multiplexed mass cytometry and comprehensive histological analysis to explore species-specific microglia heterogeneity (73–75). The study of human microglia is challenging due to the relative scarcity of non-pathological human brain tissue. However, recent studies have taken advantage of microglia isolated from post-mortem brains of donors without diagnosed neurological disease and from tissue resected during the treatment of epilepsy, brain tumours and acute ischemic stroke that is isolated from outside the area of pathology and deemed histopathologically normal.

Human microglia have not been extensively studied at the embryonic level; however, studies by Zhong et al. and Kracht et al. corroborate mouse data, suggesting there is a higher level of heterogeneity in the gestational period, which culminates in microglia acquiring a more homeostatic phenotype (76, 77). Like mice, human microglia can be differentiated based on their developmental stage, suggesting there is a progressive developmental program for human microglia development. At early gestational weeks nine through eleven microglia are enriched in genes such as *ITGAX, CLEC7A, AXL*, and *PKM*, while the later gestational weeks, fifteen to seventeen, are enriched with more canonical microglia genes (*CX3CR1, TMEM119, P2RY12*). Functions have yet to be ascribed to these phenotypes, but initial steps have been taken to compare microglia clusters to functions based on gene ontology designations (77).

To compare and contrast microglia heterogeneity within and between species, Masuda and colleagues sequenced 3,826 microglia from healthy and injured (facial nerve axotomy and cuprizone) mouse brains in addition to 1,180 human cortical microglia and 422 CD45+ cells from MS brain tissue (78). While some of the homeostatic genes translated well between mouse and human (*Cst3, P2ry12, Tmem119, Emr1*), human microglia were found to be more diverse and had clusters with higher expression of chemokines (*CCL2, CCL4*) and distinct transcription factor profiles (*EGR2, EGR3*) (78–80). This study identified homeostatic human microglia clusters with distinct profiles, but also profiles that partially overlap with those of murine microglia. This same group further explored microglia heterogeneity across 18 different species using an extensive dataset that included 1,069 human microglia. They reported significant microglial heterogeneity in humans compared to all other mammals (75).

Using both single-cell RNA sequencing and mass cytometry, Sankowski and colleagues observed both age and spatial (white vs grey matter) heterogeneity in human microglia (81). Enriched in humans is a microglia population expressing the gene *SPP1* that encodes for a proinflammatory cytokine, osteopontin (78, 82, 83). In people under the age of 30, the proportion of microglia expressing *SPP1* is negligible. However, people over the age of 50 show a five to ten-fold increase in microglia expressing *SPP1*, suggesting microglia become more inflammatory as one ages. The human age-associated proinflammatory microglia are synonymous with inflammatory profiles identified in mice. In the same study, comparisons between white and grey matter were also made, highlighting the upregulation of the MHC-II antigen presentation complex related genes, *CD68* and *HLA-DR* in the white but not the grey matter (81).

Regional variability in microglia signatures was further explored using multiplexed mass cytometry of human microglia (84). Bottcher and colleagues analyzed microglia expression across five brain regions and found two prominent patterns (84): microglia located in the temporal and frontal lobe were defined by low levels of the mannose receptor CD206, whereas those in the thalamus, subventricular zone and cerebellum had no expression of CD206. These CD206 low microglia were distinct from what are presumably perivascular macrophages that expressed high levels of CD206. This study also found that microglia express similar genes in the fresh and post mortem isolates, albeit at slightly different levels, which validates the use of post mortem tissue in the study of human microglia signatures (84).

There are some common findings concerning microglia density in both mice and humans, with higher microglia density in the white matter than grey matter. Other similarities include relatively lower densities in the cerebellar cortex compared to regions of high density, such as in midbrain and brainstem structures (2, 75, 85). Despite these commonalities, overall microglial density varies markedly between the two species. Reported differences include a higher microglial density in the frontal cortex of mice compared to humans and more microglia in the human cerebellum and hippocampus compared to mice (75). Despite these differences in density, the morphological features of microglia remain relatively similar, including branch points, terminal points and dendrite length. The functional importance of these species’ differences and the effect that these differences might have on our understanding of microglia during neurological diseases such as MS remains to be fully elucidated.

## Microglia/Macrophage Heterogeneity in the Context of CNS Autoimmunity

**Figure 4:**
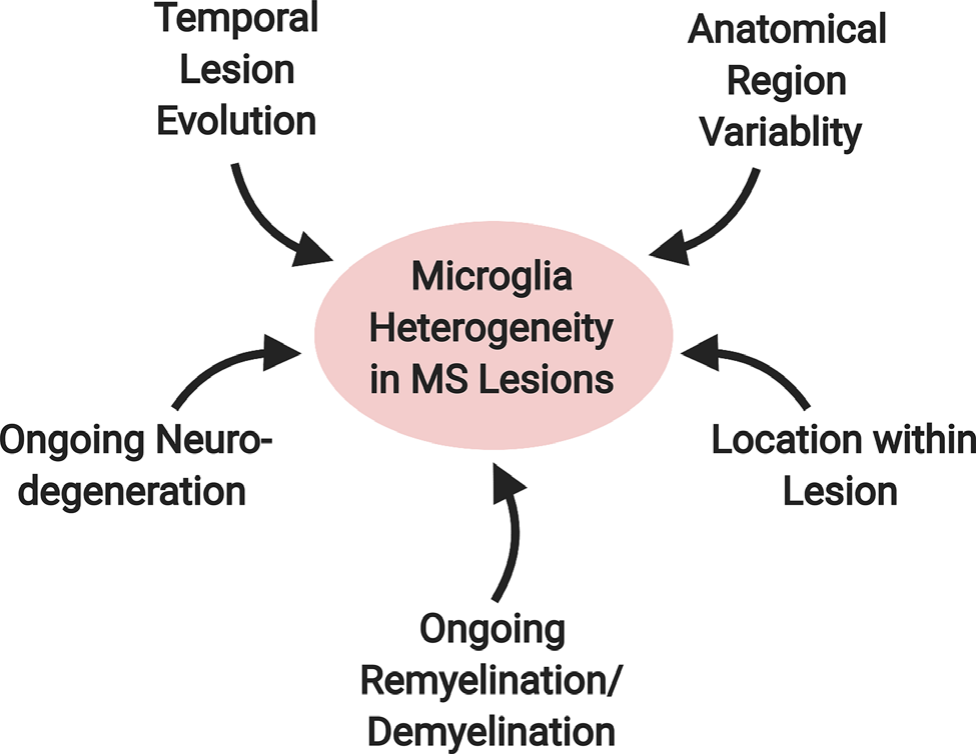
Factors contributing to microglial heterogeneity in MS lesions. A variety of factors likely contribute to the diversity of the microglial phenotype in MS. These include: 1) temporal lesion evolution, 2) ongoing neurodegeneration and 3) remyelination/demyelination in the surrounding environment, 4) location within the lesion and 5) anatomical location within the brain.

In MS, microglia and macrophage likely serve diverse roles and acquire distinct phenotypes given the variable nature of the disease. MS is characterized by demyelinating lesions along with progressive degeneration of white and grey matter (86, 87). In the active stages of MS—with the presence of new lesions—there is a dissemination of lesions in anatomical space and over time. At any given moment, a person with MS is likely to have old and new lesions in both the grey and white matter regions; these differences affect the pattern of microglial gene expression (88). The potential effects of lesion evolution on microglial/macrophage phenotypes are compounded by the presence of myelin regeneration, or remyelination. Microglia found in demyelinating and remyelinating conditions possess different phenotypes (78), with demyelination-associated microglia resembling patterns found associated with neurodegenerative disease (64). In parallel with lesion formation, MS is characterized by ongoing neurodegeneration that is often measured by advancing brain atrophy (89). Overall, the microglia and macrophage phenotypic heterogeneity and their diverse responses are likely related to temporal differences in lesion progression coupled with potential ongoing remyelination or neurodegeneration and interactions with other cell types. Regional variability in microglia phenotypes in the non-diseased state is likely to add complexity in the immune response during MS with disease characteristics convolving onto regional disease heterogeneity (**Figure 4**) (88). Animal models are designed to replicate different features of the disease to understand various aspects of MS. In this section, we focus on microglia and macrophage’s role during the autoimmune attack in the CNS. Much of what we know about the mechanism of lesion formation and evolution comes from the experimental autoimmune encephalomyelitis (EAE) model.

## Toxicity of Microglia and Monocytes During EAE

In EAE, various myelin antigens are given to a mouse in conjunction with an adjuvant to stimulate a myelin mediated autoimmune response [reviewed by (90, 91)]. Although there is variability between models, therapeutics that prevent T-cell activation or trafficking prevent EAE (92, 93). Despite T-cells’ critical role in initiating autoimmune injury, T-cells collaborate with microglia and macrophages to induce toxicity (94). For example, Heppner and colleagues used transgenic mice expressing the suicide gene thymidine kinase under the expression of the *Itgam* (*cd11b*) promoter to kill myeloid cells and found that ablation in these mice considerably repressed EAE (95). Similarly, the removal of Tak1—an NF-κB cell signalling mediator—from microglia and BAM almost completely prevented autoimmune injury (96). Selective removal of Tak1 prevented demyelination but also dramatically suppressed T-cell infiltration into the CNS suggesting that microglia or BAM regulate lymphocyte trafficking into the CNS during EAE. Monocyte-derived macrophages are also required for the autoimmune injury during EAE. Monocytes are elevated in the blood before an increase in disability and monocytes’ entry into the CNS triggers EAE progression (97, 98). Preventing monocyte entry by removing the chemokine receptor Ccr2 reduces clinical disability and toxicity in the CNS during EAE, suggesting that monocyte-derived macrophages are toxic (97, 99). Taken together, the combined efforts of microglia, BAM and monocyte-derived macrophage are required in the pathogenesis of EAE and likely contribute to lesion formation and evolution in MS.

It is still unclear whether these cells induce direct toxicity, or whether they act through indirect mechanisms. For example, while both microglia and macrophage produce reactive oxygen species (ROS) during EAE, a greater proportion of monocyte-derived macrophages express ROS producing enzymes than microglia (100). The production of ROS by phagocytes during EAE produces injury to myelin and axons alike, and can be diminished with ROS and reactive nitrogen species (RNS) scavengers (101, 102). Other direct mechanisms of toxicity by microglia and macrophage include the release of glutamate (103–105), or the expulsion of numerous potentially toxic cytokines (100, 106, 107). The toxic properties of microglia or macrophage may also be indirect. Microglia prevent the migration of infiltrating macrophages into spared tissue (8), and may also serve important “gate-keeping” functions for other leukocytes that are toxic during EAE. The roles of microglia are likely to evolve throughout the disease, as demonstrated by the finding that microglia ablation with a Csf1 inhibitor during EAE progression accelerates clinical disability (108).

## Microglia Heterogeneity During EAE

Despite the hundreds of receptor systems expressed by microglia (109), their activation and response to damage does have similarities across disease conditions. For example, microglia in an environment of amyloid induced neurodegeneration form a disease-associated microglia (DAM) (64), characterized by the downregulation of canonical microglial genes (*P2ry12/13*, *Cx3cr1*, *Tmem119*, *Cst3*) and upregulation of genes mapped to lipid metabolism pathways and phagocytosis (*Apoe*, *Lpl*, *Cst7*, *Ctsd*, *Tyrobp*, and *Trem2*). Certain genes, such as *Hexb* are stably expressed in homoeostatic microglia, DAM, and other neurological conditions (64, 110). Elements of this DAM signature were later observed in microglia activated by diverse conditions such as following white matter injury (8, 53), EAE (35), MS (78, 82), amyloid lateral sclerosis (ALS) (64, 111), ageing (53, 111), facial nerve injury (112) and cancer (81). Krasemann and colleagues analyzed gene expression patterns from microglia isolated during Alzheimer’s disease, EAE and ALS mice models and identified a common microglia response (111). The microglia response to neurodegeneration required lipid receptor and trafficking elements *Apoe* and *Trem2* under diverse disease conditions, suggesting that some aspects of microglia activation in murine disease models are conserved. Critical aspects of this microglia signature were stimulated by the injection of apoptotic neurons that were later engulfed by microglia. The typical microglia response to diverse disease conditions may be a consequence of clearing debris, dead cells or other neurodegenerative molecular patterns (113).

Despite a common microglia response to disease, there is also a diversity in the microglia response during EAE and MS (35, 78). Jordao and colleagues identified four different clusters of disease-associated microglia in mice induced with EAE (35). EAE microglia were enriched with a phenotype characterized by markers of inflammation and proliferation *Ly86, Ccl1, Cxcl10, Mki67, Ccl4*, and *Ccl5.* The *Ccl5* and *Cxcl10* provide more of an understanding of this phenotype as these chemokines aid in leukocyte recruitment, which could be a potential future avenue to explore (35). They also identified a heterogenous response by other CNS resident macrophages such as those from the leptomeninges, the perivascular space and choroid plexus (35), suggesting a CNS-wide transcriptional change during autoimmune-mediated CNS injury. Ajami and colleagues also identified a population of CNS associated macrophages enriched in expression of diverse cytokines that were not found in healthy mice and peaked during symptomatic EAE (114).

Diverse populations of microglia were also found in the MS brain (78). Three subsets of MS-specific microglia were identified. Subsets were enriched for *SPP1* or *CD74*, which also defined microglia from mice given the demyelinating agent cuprizone and isolated under either demyelinating or remyelinating conditions, respectively (78). The transcriptional signature of microglia may one day be used to determine whether a lesion is demyelinating or remyelinating. The MS lesion exhibits marked diversity: Park and colleagues used imaging mass cytometry to examine the heterogeneity of CNS-associated macrophages and found that their diversity could be stratified based on their relative location within the MS lesion with enriched lysosomal LAMP1 or receptor tyrosine kinase MERTK expression on myeloid cells located at the lesion rim (115, 116). Taken together, microglia initiate certain conserved activation patterns in diseased conditions but also, microglia exhibit several unique phenotypes likely reflecting their local environment. Understanding the function and ubiquity of disease-specific microglia phenotypes will provide a greater understanding of neurological diseases.

## Monocyte Heterogeneity During EAE

Monocyte diversity in the CNS similarly increases during autoimmunity. Ajami and colleagues identified five subsets of monocytes in the CNS that changed their expression profile throughout EAE (114). Peak EAE is defined by the simultaneous expression of three or four different cytokines in a given cell, not found in homeostatic subsets. By comparing the surface markers of blood-derived myeloid cells to the CNS-resident macrophage, Ajami and colleagues identified a new cell surface marker, Cd49e—or α5 integrin—that is upregulated by infiltrating monocytes (114). Treatment with antibodies that blocked Cd49e delay the onset and reduced the severity of EAE. Using single-cell RNA sequencing, Giladi also examined monocyte and monocyte-derived macrophage diversity during EAE finding eight distinct populations (117). Using antibodies against Ccr2 to ablate monocytes and reduce EAE severity, Giladi and colleagues identified two distinct monocyte populations that were selectively lost, and presumably are pathogenic given their association with disease conditions (117). Surprisingly, monocyte depletion resulted in minor changes to other immune cells suggesting monocytes may be pathogenic due to direct cytotoxicity. Given the toxic role of monocyte-derived macrophages, understanding monocyte diversity—with particular focus on pathogenic populations and how they traffic into the CNS—will lead to new macrophage focused therapies.

## Microglia/Macrophage Populations During Remyelination

Myelin injury is a crucial attribute of demyelinating diseases such as MS, but so is the regeneration of myelin, or remyelination. For people with MS, remyelination occurs, but it is highly variable and prone to failure (118–122). Remyelination can restore lost behaviour due to myelin injury (123) and protects axons from degeneration (124)—which causes irreversible harm that is thought to contribute to ongoing progression. Indeed, promoting remyelination spares axons and improves functional recovery following EAE (125). For these reasons, finding therapeutic agents that promote remyelination is an exciting new avenue to treat MS. Several clinical trials are ongoing but no therapies have been approved as of yet (122, 126). Remyelination requires a favourable immune response from macrophage/microglia to clear inhibitory myelin debris and secrete growth factors and cytokines, such as Igf1 and activin-A, that regulate remyelination and the extracellular matrix (127–129). Despite the many benefits of the immune response to remyelination, there are only a few strategies that focus on improving the immune response as a means to boost remyelination (130–133). The paucity of immune-boosting targets in MS likely reflects the challenges of promoting immune activities because there are numerous immune-mediated mechanisms of neurotoxicity that could potentially be triggered.

Pioneering work by Miron and colleagues demonstrate that microglia/macrophages take on a proinflammatory signature early after demyelination that promotes OPC proliferation (134). These proinflammatory macrophage/microglia secrete cytokines such as Il1β and Tnf, which stimulate OPC survival and proliferation (135, 136). The proinflammatory microglia/macrophage then transition to an immunoregulatory phenotype (134). Ablation of these immunoregulatory immune cells contributes to remyelination failure suggesting that the transition from the proinflammatory state to an immunoregulatory one is an important step during remyelination. Unknown from this work is whether microglia or macrophage express these proinflammatory or immunoregulatory factors. Research from our group shows that the classic proinflammatory (iNos) and immunoregulatory markers (Arg-1) used by Miron and colleagues are not expressed by microglia following LPC mediated demyelination of the spinal cord (8). The proinflammatory and immunoregulatory phenotypes described by Miron and colleagues may therefore be attributed to blood-derived macrophages. Recently, Lloyd and colleagues investigated how microglia/macrophage transition from a proinflammatory to an immunoregulatory phenotype (127). Surprisingly, this transition required necroptosis, a form of programmed necrosis. Inhibiting necroptosis stalled remyelination and maintained high levels of proinflammatory microglia/macrophages, suggesting that necroptosis regulates the shift away from the proinflammatory phenotype.

While microglia and macrophages can take on proinflammatory and immunoregulatory phenotypes, new deep phenotyping of immune cells suggests that there are more diverse immune states after demyelination. We identified three distinct microglia phenotypes by isolating microglia following LPC-mediated demyelination of the spinal cord and conducting single-cell RNA sequencing (8). Microglia were isolated five days after LPC demyelination–a time point before remyelination characterized by OPC recruitment (137–139). We found that most activated microglia were enriched for *Spp1*, or osteopontin, *Apoe*, and *Cd74* (8). These genes are commonly expressed in microglia within the diseased, neurodegenerative CNS (64) and may, therefore, reflect microglia that are responding to damage or neurodegenerative molecular patterns (113). We also found a population of microglia enriched in interferon associated genes such as *Ifit3*, *Irf7*and *Ifitm3* as well as a third population likely reflecting proliferative microglia (8). At seven days after LPC demyelination of the corpus callosum, Hammond and colleagues similarly used single-cell RNA sequencing and identified similar populations of microglia, suggesting the microglial response may be consistent between these regions (53). Yet to date, none of the deep sequencing studies to date have investigated how microglia or macrophages change throughout the continuum of remyelination. This work could identify yet more states of immune cell activity.

The tools for differentiating microglia and macrophages are relatively novel and understanding the regenerative and neurotoxic aspects of these cell types is an area of research still in its infancy. Remyelinating models are valuable tools to understand the beneficial aspects of the immune response. After all, remyelination is perhaps the clearest example of regeneration in the CNS and likely resembles regenerative processes in other tissues that also depend on a tightly regulated inflammatory response. Presumably, the immune cell phenotype will inform its function; therefore, identifying remyelination associated microglia and macrophage phenotypes are vital. Identifying immune cell phenotypes may also provide new biomarkers for remyelination. In MS, microglia/macrophages make up the majority of immune cells within the lesion (140) and MS lesions classification often relies on the presence and location of activated microglia/macrophage (141). However, activated microglia/macrophages are enriched during ongoing CNS injury (142) and can present during remyelination (143, 144). The accumulation of microglia/macrophage is, therefore, not a sensitive predictor of injury or regeneration. Given that microglia and macrophage are highly plastic and take on a unique cell state in response to diverse disease conditions, these cell states may indicate the stage or relative toxicity of the immune response. Indeed, the phenotype of microglia during active demyelination is distinct from microglia during remyelination (78).

## Age-Associated Remyelination Decline Involves Impaired Microglia/Macrophage Response

It has been known for almost three decades that the efficiency of remyelination declines with ageing (145, 146). Given that remyelination protects axons from degeneration (124), preventing remyelination decline due to ageing may slow MS neurodegeneration. Mechanisms underlying this age-related impairment have been attributed to both CNS-intrinsic and extrinsic factors (147). For example, extrinsic factors such as the inadequate clearance of myelin debris in aged mice are restored by a more youthful peripheral immune response (148). Interestingly, the ageing demyelinated lesion increases in stiffness, potentially due to the extracellular matrix remodelling functions of aged microglia/macrophage (149), which impairs remyelination (150).

As activated microglia and infiltrating macrophages play an essential role in remodelling the lesion microenvironment, the changes these cell types undergo with ageing have a direct impact on the age-related impairment in remyelination efficiency. One of the first studies to document this link observed a delay in the expression of several essential growth factors following demyelination in ageing animals (151). This alteration in Pdgfa, Tgf-β, and Igf1 was associated with a delay in recruiting macrophages and microglia to lesions in ageing rats (152). In addition to this dysregulation in growth factor kinetics, lesions from ageing rodents displayed an accumulation of inhibitory myelin debris, suggesting that macrophages’ and microglia’s phagocytic capacity becomes impaired with ageing (148). Several studies have now highlighted a deficiency in the ability of ageing microglia and macrophages to phagocytose myelin debris (130, 133, 153). These alterations have been attributed to a disruption in retinoid X receptor signalling and a decrease in the expression of the scavenger receptor Cd36 (130, 133). In addition to deficiencies in the initial engulfment of myelin debris, another group identified disruptions in the lysosomal processing and subsequent cholesterol efflux of ingested myelin (71, 131). Accumulation of lysosomal inclusions and cholesterol crystals in ageing microglia resulted in inflammasome signalling and proinflammatory cytokine expression, resulting in a lesion microenvironment not conducive to efficient regeneration.

Due to difficulties distinguishing microglia from monocyte-derived macrophages within the lesion, no studies to date have been able to assign intralesional functional differences between these two cell populations with ageing. The advent of phenotypic markers and genetic fate-mapping strategies to distinguish these two populations opens up a promising new avenue of inquiry (110, 154, 155). Circumstantially, it has been documented that the ageing process manifests differently in microglia compared to monocyte-derived macrophages. As microglia are self-renewing cells within a CNS microenvironment that accumulate myelin fragments and protein aggregates with advancing age, they assume a senescent phenotype that is “primed” (71, 156). Single-cell sequencing of microglia from the ageing brain shows the expansion of two different clusters that upregulate several inflammatory signals such as *Ccl4, Il1b*, as well as several interferon-response genes (53).

In contrast, ageing monocyte-derived macrophages display an impairment in producing a functional proinflammatory cytokine response when stimulated with potent activating agents such as LPS (157). As the half-life of circulating monocytes in humans is approximately 71 h, it is postulated that the age-related changes in monocyte-derived macrophages manifest at earlier stages in monocyte development, such as at the level of the hematopoietic stem cell (158, 159). In addition to differences in the manifestations of ageing between microglia and monocyte-derived macrophages, it is now appreciated that microglia from diverse regions within the CNS also age differently (68). Future studies using single-cell sequencing and genetic fate-mapping to dissect microglial and macrophage transcriptional and functional heterogeneity within lesions and in the context of ageing will be essential to establish better how best to target these cells therapeutically and promote myelin regeneration.

## Conclusion

We are at the dawn of a new era in recognizing microglia heterogeneity. Research is accelerating to identify microglial phenotypes throughout development and disease. Work must continue to expand upon our understanding of the gene and protein expression of microglia during development, throughout life, at different stages of disease and in different spatial locations relative to damage as this research will advance our knowledge of microglial functions and the interactions between microglia and other cell types.

Defining microglia will provide new cellular and phenotypic markers that can be used to detect and manipulate microglia phenotypes in MS and other neurological conditions. As the primary innate immune cells of the brain and spinal cord, microglia are uniquely positioned to both exacerbate the injury and be neuroprotective or even reparative. Still, with only recently available tools to target microglia directly, much work remains to define microglia function in different conditions. In the field of MS, we must still differentiate the contributions of microglia, infiltrating macrophages and BAM during remyelination, progression and throughout autoimmune injury. The next frontier will be to resolve the functions of these different microglia phenotypes.

Important questions remain: are there neuroprotective or neurotoxic microglial phenotypes? If so, what factors promote these phenotypes? Can they be targeted therapeutically? The availability of serum-free cell culture models for murine (160) and human cells (161–164) will support the functional analyses of distinct microglial phenotypes. Newer single-cell sequencing modalities such as IN-seq (165) or CITE-seq (166) allow protein markers to be overlaid onto single-cell sequencing defined immune cell phenotypes, permitting comparisons of cellular signalling or state to immune phenotypes. Strategies such as Tox-seq are also available to differentiate one function—ROS production—and overlay this function onto immune cell clusters. Bioinformatic tools such as NicheNet (51) and CellPhoneDB (52) provide a way to identify new receptor-ligand pairs from single-cell RNA sequencing data, which will serve as the starting point to dissect intercellular communications between CNS macrophages and their surrounding cellular niche that can then be studied *in vitro* and *in vivo*. With these and other available tools, it will be possible to dissect functionally distinct microglial and macrophage phenotypes so that they can be manipulated in MS and other neurological conditions. This ability offers the potential to harness the immune system’s capabilities and bias the CNS lesion environment towards protection and repair rather than damage.

## Author Contributions

SZ, KR, NM, BK, LH, and JP drafted and reviewed the manuscript. MB and SZ constructed the figures. JP supervised the drafting of this manuscript. All authors contributed to the article and approved the submitted version.

## Funding 

SZ is funded by a Masters NSERC fellowship. KR is funded by the MS Society of Canada. MB is funded by an Alberta Innovates studentship. This work was funded by operating grants held by JP from CIHR, University of Alberta, Brain Canada and the Azrieli Foundation. Figures were developed with bio Render.

## Conflict of Interest

The authors declare that the research was conducted in the absence of any commercial or financial relationships that could be construed as a potential conflict of interest.
